# Virulence determinants associated with the Asian community-associated methicillin-resistant *Staphylococcus aureus* lineage ST59

**DOI:** 10.1038/srep27899

**Published:** 2016-06-14

**Authors:** Min Li, Yingxin Dai, Yuanjun Zhu, Chih-Lung Fu, Vee Y. Tan, Yanan Wang, Xing Wang, Xufen Hong, Qian Liu, Tianming Li, Juanxiu Qin, Xiaowei Ma, Jingyuan Fang, Michael Otto

**Affiliations:** 1 Department of Laboratory Medicine, Renji Hospital, School of Medicine, Shanghai Jiaotong University, Shanghai, China; 2 Pathogen Molecular Genetics Section, Laboratory of Bacteriology, National Institute of Allergy and Infectious Diseases, The National Institutes of Health, Bethesda, Maryland, USA; 3 Department of Laboratory Medicine, Shanghai Children’s Medical Center, School of Medicine, Shanghai Jiaotong University, Shanghai, China; 4 Department of Gastroenterology and Hepatology, Renji Hospital, School of Medicine, Shanghai Institute of Digestive Disease, Shanghai Jiaotong University, Shanghai, China

## Abstract

Understanding virulence is vital for the development of novel therapeutics to target infections with community-associated methicillin-resistant *Staphylococcus aureus* (CA-MRSA), which cause an ongoing epidemic in the United States and are on a global rise. However, what defines virulence particularly of global CA-MRSA lineages is poorly understood. Threatening a vast population, the predominant Asian CA-MRSA lineage ST59 is of major epidemiological importance. However, there have been no molecular analyses using defined virulence gene deletion mutants in that lineage as of yet. Here, we compared virulence in skin, lung, and blood infection models of ST59 CA-MRSA isolates with geographically matched hospital-associated MRSA isolates. We selected a representative ST59 CA-MRSA isolate based on toxin expression and virulence characteristics, and produced isogenic gene deletion mutants of important CA-MRSA virulence determinants (α-toxin, PSM α, Agr) in that isolate for *in-vitro* and *in-vivo* analyses. Our results demonstrate strongly enhanced virulence of ST59 CA-MRSA over hospital-associated lineages, supporting the notion that enhanced virulence is characteristic for CA-MRSA. Furthermore, they show strong and significant contribution of Agr, α-toxin, and PSMα to pathogenesis of ST59 CA-MRSA skin, lung, and blood infection, emphasizing the value of drug development efforts targeted toward those virulence determinants.

Traditionally, MRSA clones exclusively caused hospital-associated infections in predisposed individuals. In contrast, the recently emerged community-associated (CA)-MRSA clones combine methicillin resistance and increased virulence potential in a manner that allows the infection of otherwise healthy people outside of hospital settings^1^. Most CA-MRSA infections present as moderately severe skin and soft tissue infections (SSTIs), but CA-MRSA may also cause more severe and fatal infections such as necrotizing pneumonia^2^. The first CA-MRSA cases were observed in the late 1990s in the Midwestern U.S.^3^, and especially the U.S. has since experienced a severe and ongoing CA-MRSA epidemic due to the highly virulent CA-MRSA clone USA300^4^.

The basis of virulence in CA-MRSA is not yet completely understood, but it appears to be a combination of genetic adaptations that result in a balanced “compromise” between enhanced virulence and simultaneous maintenance of methicillin resistance. The latter is linked to the low-fitness cost, small SCC*mec* elements, which are characteristic for CA-MRSA and contain the methicillin resistance genes^5^. To explain the enhanced virulence potential of CA-MRSA, two not mutually exclusive hypotheses were developed^1^. One focuses on the acquisition of mobile genetic elements, in particular the prophage harboring the Panton-Valentine leukocidin (PVL)^6^. The other hypothesis explains enhanced virulence by increased expression of cytolytic toxins, including α-toxin and phenol-soluble modulins (PSMs) α, whose genes are present in virtually all *S. aureus* strains^7^,^8^,^9^.

Importantly, our notions about the basis of virulence of CA-MRSA are based almost exclusively on studies of clone USA300. However, globally, CA-MRSA infections are due to different lineages with specific lineages frequently dominating in a given geographic location. The most widespread CA-MRSA clone in large parts of Asia is sequence type (ST) 59^10^. Considering the high population in the areas where ST59 is the predominant CA-MRSA clone, this clone may threaten a greater number of people than USA300. However, with few exceptions^11^,^12^,^13^, global CA-MRSA clones have not been investigated on the molecular level, such as by using gene deletions in virulence determinants.

Here we performed detailed molecular investigation of ST59 CA-MRSA. We compared virulence to geographically matched HA-MRSA clones and selected a representative clone based on virulence and virulence factor expression to produce isogenic gene deletion mutants in premier CA-MRSA virulence determinants, which we analyzed using animal models of skin, lung, and blood infection as the most prominent CA-MRSA disease manifestations.

## Results

To investigate the virulence characteristics of the CA-MRSA ST59 lineage, we analyzed all infectious ST59 CA-MRSA isolates (n = 25) obtained in 2014 at Renji hospital, Shanghai, a large teaching hospital with more than 20,000 admissions per day from the entire Shanghai metropolitan area. We compared them with all HA-MRSA isolates of ST5 (n = 141) and ST239 (n = 103) obtained at the same hospital in the same time. ST5 and ST239 are reportedly the most frequent HA-MRSA types in China^14^, which was also the case in our hospital: ST5 caused 48% and ST239 44% of total HA-MRSA infections between 2005 and 2014 at Renji hospital.

**Figure 1:**
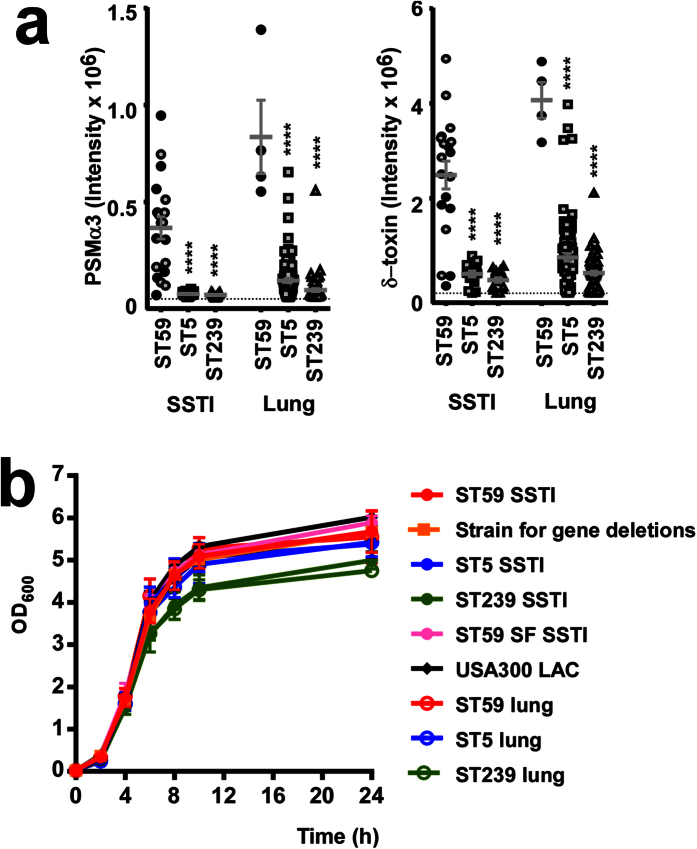
Selection of representative strains by determination of PSMα3 and δ-toxin production levels. (**a**) Relative PSMα3 and δ-toxin amounts in all ST59 CA-MRSA, ST5 HA-MRSA, and ST239 HA-MRSA isolates from skin and lung infections obtained from Renji hospital in 2014. Isolates selected for further study are shown with filled symbols. The peptide toxins were measured in cultures grown in TSB for 8 h by reversed-phase high-pressure chromatography/ion spray mass spectrometry. Comparisons are by 1-way ANOVA with Dunnett’s post-test versus ST59. ****p < 0.0001. Error bars show the standard error of the mean (±SEM). (**b**) Growth curves in TSB of all selected strains.

To obtain a representative and experimentally manageable subset of isolates, we first determined production levels of PSMα3 in all isolates from SSTI and lung infections, which accounted for the vast majority of infections by those isolates (ST59: SSTI 76%, lung infections 16%; ST5: SSTI 11%, lung infections 66%; ST239: SSTI 15%; lung infections 62%) (Fig. 1a). PSMα3 is the most potent PSM in *S. aureus* and a widespread *S. aureus* virulence determinant^7^,^8^. Together with δ-toxin, whose relative production levels reflected those of PSMα3 (Fig. 1a), it is also a direct indicator of the activity of the main global virulence regulator system Agr^15^,^16^. Isolates for further *in-vitro* and *in-vivo* experiments, which showed comparable *in-vitro* growth patterns (Fig. 1b), were selected so that average PSMα3 production levels were close to those of all isolates within a group (Fig. 1a). Thus, average virulence potentials of selected subsets should adequately reproduce those of the entire groups.

**Figure 2:**
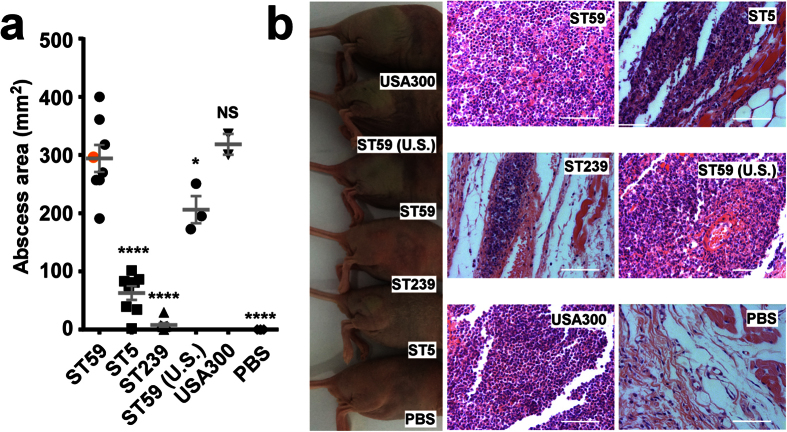
Comparison of ST59 CA-MRSA strains with geographically matched HA-MRSA and reference strains in an abscess infection model. (**a**) Mice were injected subcutaneously with ~10^7^ CFU of 8 selected, representative isolates each of ST59, ST5 and ST239; 3 U.S. ST59 SSTI isolates (one mouse per isolate) and abscess areas were measured at day 2 after infection. Control animals (n = 3) received only sterile PBS. Values for USA300 are based on two mice infected with strain LAC. The data point corresponding to the strain selected for deletion mutant construction is represented in orange color. Comparisons are by 1-way ANOVA with Dunnett’s post-test versus ST59. *p < 0.05; ****p < 0.0001. Error bars show ±SEM. (**b**) Representative abscesses and histological results (H&E stain). Scale bars are 50 μm. Note pronounced infiltration of leukocytes (purple) in ST59 and USA300-infected tissue. The skin surface is located toward the left side of the pictures.

**Figure 3:**
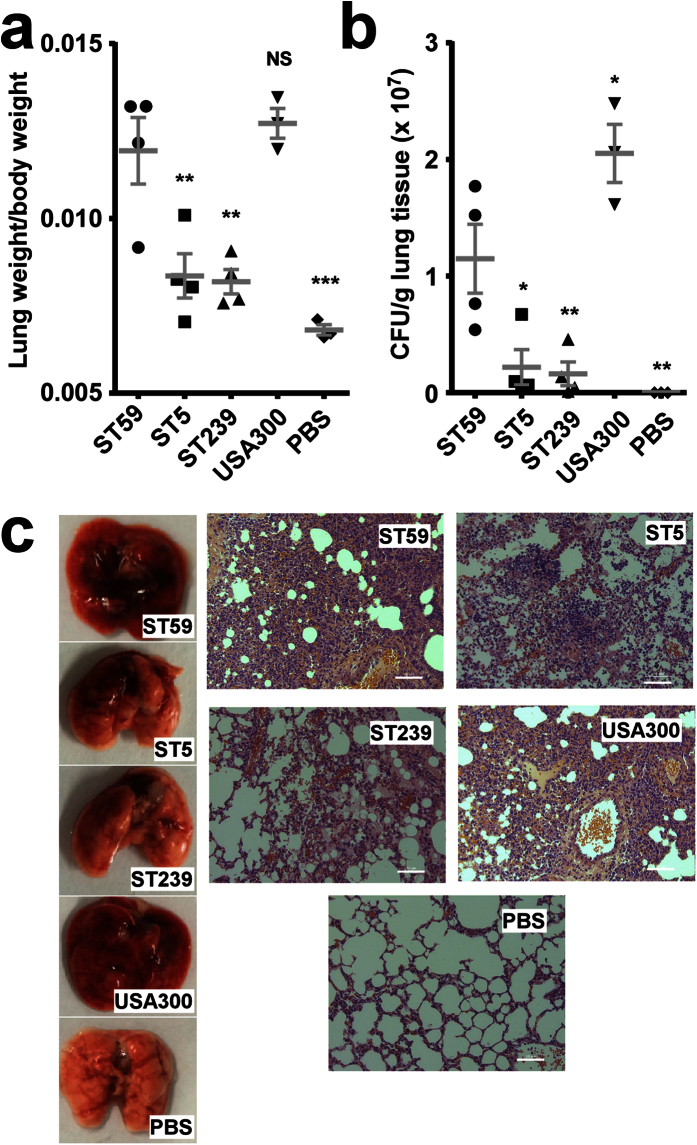
Comparison of ST59 CA-MRSA strains with geographically matched HA-MRSA and reference strains in a lung infection model. (**a,b**) 4 × 10^9^ CFU was pipetted into the nares of mice (4 selected, representative isolates each of ST59, ST5 and ST239; Values for USA300 are based on three mice infected with strain LAC). Control animals (n = 3) received only sterile PBS. Lung weight, body weight (**a**), and CFU (**b**) were measured after euthanizing mice 48 h after infection. Comparisons are by 1-way ANOVA with Dunnett’s post-test versus ST59. *p < 0.05; **p < 0.01; ****p < 0.0001. Error bars show ±SEM. (**c**) Representative lungs and histological results (H&E stain) from infected animals. Scale bars are 50 μm. Note lung enlargement and hyperemia, infiltration of erythrocytes (orange) and leukocytes (purple), in ST59 and USA300-infected lungs.

First, we analyzed whether CA-MRSA ST59 isolates have increased virulence, as reported for USA300 and other CA-MRSA clone^1^,^7^,^11^,^17^, in skin and lung infection models as compared to the HA-MRSA isolates. Notably, we only used isolates that originated from the corresponding type of human infection. In the skin infection model, abscesses caused by ST59 isolates were significantly larger than those caused by ST5 and ST239 isolates (Fig. 2a,b). Histological examinations showed more extensive inflammation with leukocyte infiltration, destruction of the skin structure, and inflammation that extended to dermal tissue (Fig. 2b). In the lung infection model, lung pathology as measured by lung weight and bacterial load was significantly more pronounced in ST59 than in ST5 and ST239 isolates (Fig. 3a,b), which was reflected by macroscopic lung examination and histopathology results (Fig. 3c). Mice infected with ST59 showed extensive inflammation with disruption of pulmonary architecture, hemorrhagic infiltration, and influx of leukocytes. Notably, pathology in skin and lung infection models of ST59 isolates reached levels similar to those caused by the USA300 clone LAC (Figs 2 and 3). These findings demonstrate pronounced virulence of ST59 CA-MRSA isolates.

**Figure 4:**
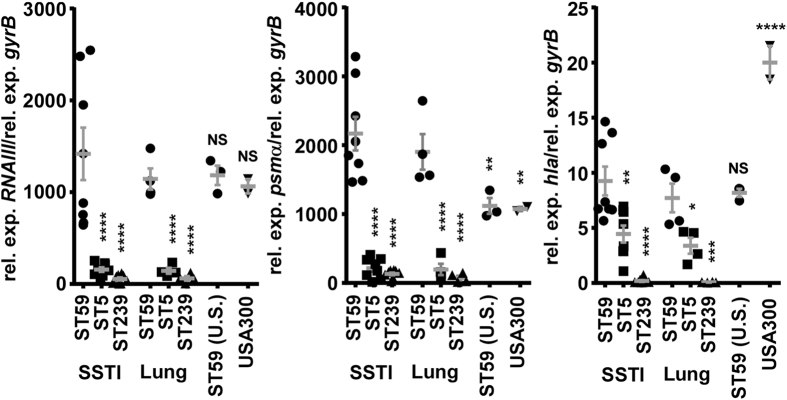
Expression of *psm*α operon (encoding PSMα peptides), *hla* (α-toxin), and *agr.* Expression levels were determined by qRT-PCR at stationary growth phase (8 h) during growth in TSB. Rel. exp., relative expression. ***p < 0.001; ****p < 0.0001. Comparisons are between all SSTI strains (ST59, ST5, ST239, USA300, ST59 U.S.) or between all lung infection isolates (ST59, ST5, ST239). *p < 0.05; **p < 0.01; ***p < 0.001; ****p < 0.0001 (1-way ANOVA with Dunnett’s post-test vs. ST59). NS, not significant (p ≥ 0.05). Error bars show ±SEM.

To understand the basis of virulence in ST59 CA-MRSA, we first considered the most important reported mobile genetic element-encoded virulence determinants of CA-MRSA, PVL and the arginine mobile genetic element (ACME)^6^,^18^,^19^. PVL genes were only present in a minority of our 2014 ST59 CA-MRSA isolates (36%), indicating that PVL is not a determining general factor of enhanced virulence of ST59 CA-MRSA. Similarly, ACME, shown to contribute to the virulence of USA300^18^, was absent from the ST59 isolates. Therefore, we hypothesized that enhanced virulence of ST59 CA-MRSA is due to increased expression of core-genome encoded virulence determinants. We determined expression levels of the two genome-encoded toxins most frequently linked to CA-MRSA virulence, α-toxin (Hla) and PSMα peptides^8^,^9^, and of the Agr system controlling expression of these toxins^20^. Expression levels of the *hla* and *psm*α genes as well as that of RNAIII, the intracellular effector of Agr^21^, were significantly increased in the ST59 versus ST5 and ST239 isolates (Fig. 4), which is in accordance with the observed significantly increased amounts of PSMα3 and δ-toxin in culture filtrates (Fig. 1a). These results suggest that α-toxin, PSMα, and Agr expression contribute to the observed increased virulence potential of the ST59 CA-MRSA isolates.

**Figure 5:**
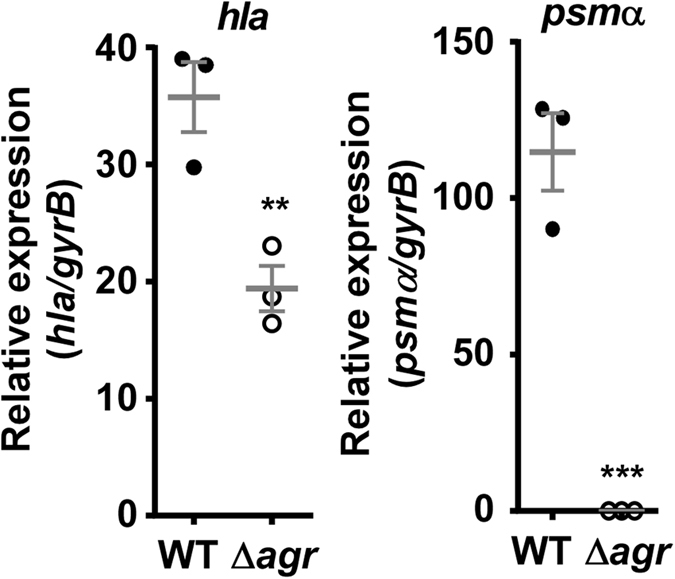
Agr controls *hla* and *psm*α expression in ST59 CA-MRSA. Shown are qRT-PCR measurements at 4 h of growth in TSB of wild-type ST59 CA-MRSA and isogenic *hla* and *psm*α deletion mutants. Comparisons are by unpaired t-tests. **p < 0.01; ***p < 0.001. Error bars show ±SEM.

**Figure 6:**
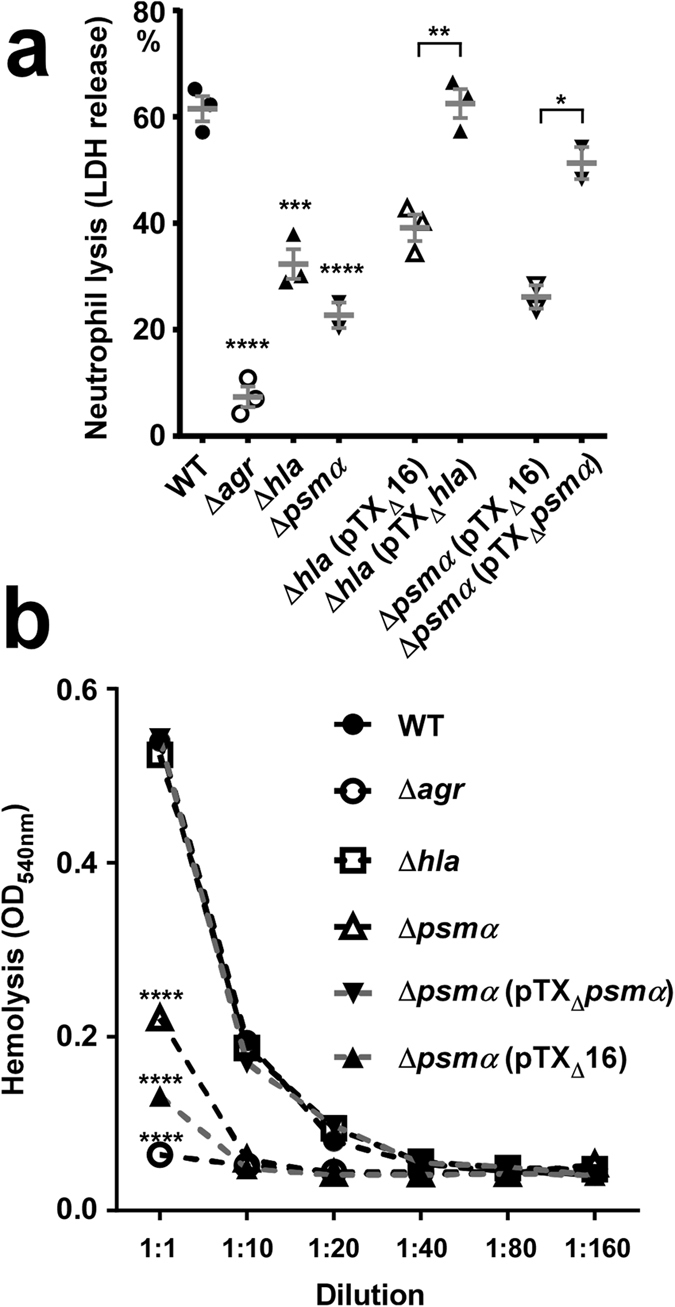
PSMα, α-toxin and Agr impact the capacity of ST59 CA-MRSA to lyse human red and white blood cells. (**a**) Lysis of human neutrophils by bacteria. Bacteria were grown to late logarithmic growth phase and neutrophils were incubated at a 10:1 ratio. (**b**) Lysis of human erythrocytes by culture filtrates of bacterial cultures at increasing dilutions. (**a,b**) *p < 0.05; **p < 0.01; ***p < 0.001; ****p < 0.0001; for two groups comparisons are by unpaired t-tests (for comparison of the plasmid-based expression versus with the corresponding strain harboring plasmid control); for more groups comparisons are by 1-way or 2-way ANOVA with Dunnett’s post-test. In (**b**), only statistics for the 1:1 dilution are shown, but differences were significant down to a dilution of 1:20 (for Δ*hla* and Δ*psm*α vs. WT) or 1:40 (for Δ*psm*α pTX_Δ_*psm*α vs. Δ*psm*α pTX_Δ_16). (**a,b**) Error bars show ±SEM.

To directly analyze the importance of PSMα peptides, α-toxin, and Agr in the Chinese ST59 CA-MRSA strain background, we produced isogenic gene deletion mutants in a representative isolate (as judged by virulence in the skin infection model, Fig. 2a). We first confirmed the impact of Agr regulation on *hla* and *psm*α expression, which was much stronger for *psm*α, most likely owing to the described exceptionally direct mechanism of *psm*α regulation by Agr^16^ (Fig. 5). Then, we analyzed lysis of human neutrophils and erythrocytes as key mechanisms of aggressive *S. aureus* virulence. Agr, α-toxin, and PSMα peptides had a significant effect on neutrophil lysis (Fig. 6a). The effect of Agr was particularly strong, in accordance with the fact that Agr controls expression of both *hla* and *psm*α^8^,^22^. Erythrocyte lysis was strongly reduced in the *agr* and *psm*α deletion mutants, while deletion of *hla* showed no significant effect, in accordance with the known insensitivity of human erythrocytes to α-toxin^23^ (Fig. 6b). These findings attribute strong importance to α-toxin and PSMα peptides in lysing neutrophils and to PSMα peptides in lysing erythrocytes in the ST59 genetic background, as well as to Agr control in both these phenotypes.

**Figure 7:**
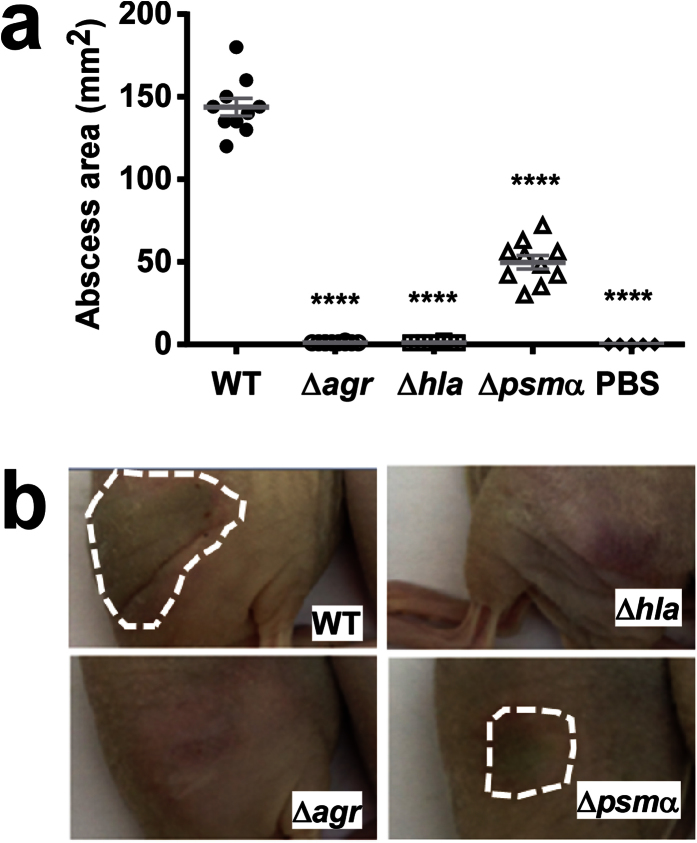
Impact of *psm*α, *hla* and *agr* on virulence of ST59 CA-MRSA in skin infection. **(a)** Skin infection model. Ten mice per group were injected subcutaneously with ~10^7^ CFU and abscess areas were measured at day 2 after infection. ****p < 0.0001 (1-way ANOVA with Dunnett’s post-test vs. WT). Error bars show the standard error of the mean (SEM). (**b**) Representative abscesses.

**Figure 8:**
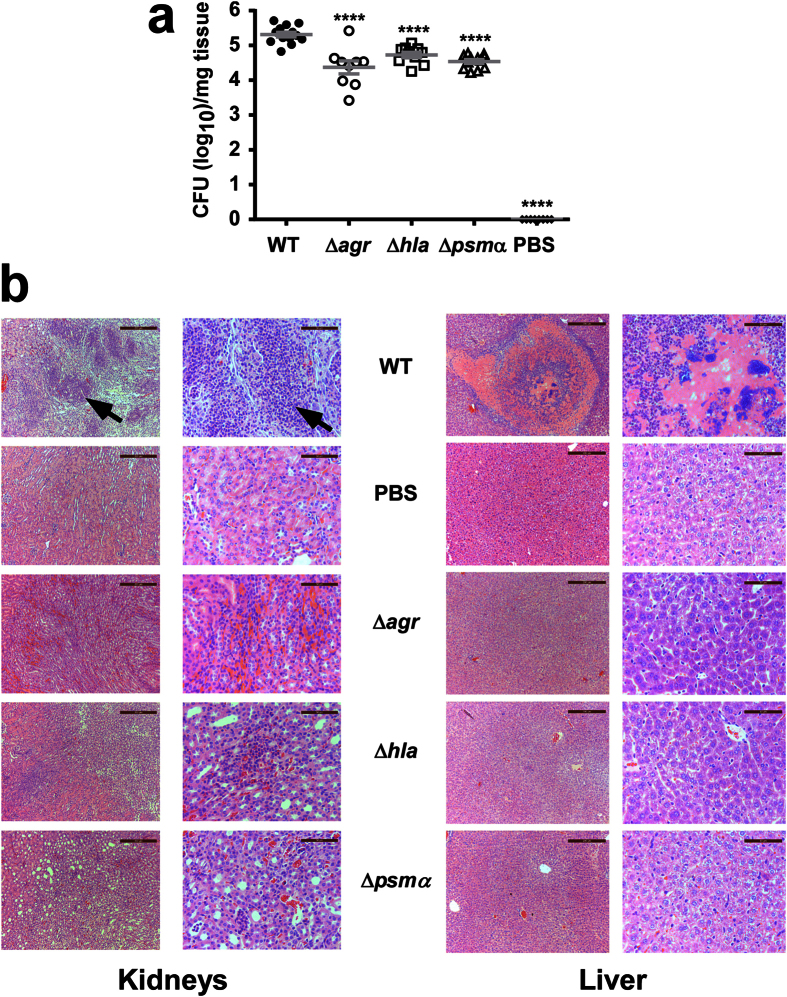
Impact of *psm*α, *hla* and *agr* on virulence of ST59 CA-MRSA in blood infection. **(a)** Ten mice per group were injected intro the retro-orbital vein with ~10^7^ CFU and CFU in kidney tissue were measured at day 4 after infection. ****p < 0.0001 (1-way ANOVA with Dunnett’s post-test vs. WT). Error bars show ±SEM. **(b,c)** Histological evaluation of kidneys and livers. Pictures show representative microscopic images of kidney and liver tissue at lower (left, scale bar 200 μm) and higher (right, scale bar 50 μm) magnification. In kidney samples, note abscess formation (arrow top left) and excessive infiltration of inflammatory cells (arrow top right) in mice infected with the WT strain, while these were largely absent from control mice and mice infected with the isogenic *agr*, *hla*, and *psm*α mutant strains. In liver samples, note necrosis and excessive infiltration of inflammatory cells of mice infected with the WT strain, while these were largely absent from control mice and mice infected with the mutant strains.

**Figure 9:**
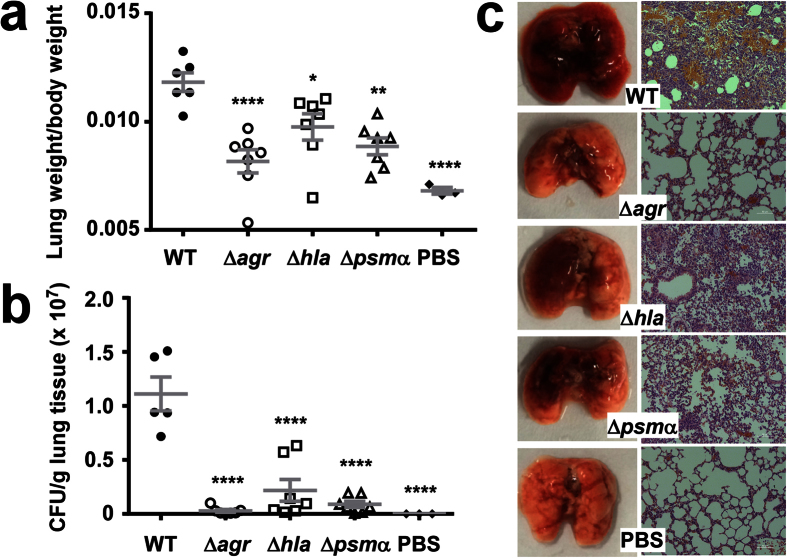
Impact of *psm*α, *hla* and *agr* deletion on virulence of ST59 CA-MRSA in lung infection. (**a,b**) 4 × 10^9^ CFU was pipetted into the nares of mice (n = 7). Control animals (n = 3) received only PBS. One mouse in the WT group died 24 h after infection. The lung weight, body weight, and CFU of all other mice were measured after euthanizing the mice 48 h after infection. *p < 0.05; **p < 0.01; ****p < 0.0001 (1-way ANOVA with Dunnett’s post-test vs. WT). Error bars show ±SEM. **(c)** Macroscopic and histological (H&E stain) examination of lungs from representative infected animals. Scale bars are 50 μm. Note the reduction of enlargement and hyperemia, and infiltration of erythrocytes (orange) and leukocytes (purple), in the mutant versus WT-infected lungs.

Then, we analyzed the impact *agr*, *hla*, and *psm*α have on virulence in murine skin, lung and blood infection models, using the isogenic gene deletion mutants. In the skin infection model, the *agr* and *hla* deletion mutants did not produce any abscesses, while abscesses caused by the *psm*α mutant were strongly reduced as compared to those caused by the wild-type strain (Fig. 7a,b). In the bacteremia model, bacterial CFU in the kidneys were significantly reduced, by ~1 log, in all three mutants compared to the wild-type-infected mice (Fig. 8a). Histological examination of liver and kidney tissue showed abscesses and pronounced infiltration of inflammatory cells in wild-type-infected mice, while these signs were largely absent from mice infected with the three mutant strains (Fig. 8b). In the lung infection model, lung per body weight rations and bacterial loads were significantly reduced in mice infected with any of the three mutants as compared to mice infected with the wild-type strain (Fig. 9a,b). Morphological examination of lungs showed reduction of enlargement and hyperemia, and histological examination of lung tissue reduced infiltration of erythrocytes and leukocytes in the mutant versus wild-type-infected lungs (Fig. 9c). Interestingly, Agr and PSMα appeared to have a stronger impact on lung disease-related phenotypes than α-toxin. Altogether, significantly reduced pathology of the mutants in all three models demonstrated that α-toxin, PSMα peptides and Agr have a significant impact on these most important infection types caused by ST59 CA-MRSA.

Finally, we also investigated how the Chinese ST59 CA-MRSA isolates relate in virulence factor expression and virulence to CA-MRSA ST59 SSTI isolates obtained from the U.S. In the skin infection model, the Chinese isolates caused slightly, but significantly larger abscesses than the U.S. isolates, and showed similar pathology upon histological examination (Fig. 2a,b). Furthermore, *psm*α, but not *hla* or RNAIII expression, was significantly higher in the Chinese ST59 than in the U.S. isolates (Fig. 3). These findings are in accordance with the specific role of PSMα peptides as virulence determinants in skin infections, which was shown by animal and epidemiological studies^8^,^24^,^25^. Furthermore, they suggest that – while ST59 is a generally virulent CA-MRSA lineage – the Chinese ST59 isolates developed even further enhanced virulence due to increased expression of the *psm*α genes.

## Discussion

The analysis of virulence determinants in CA-MRSA clones is an important prerequisite for the prioritization of targets for virulence-targeted drug development. The emergence of CA-MRSA clones with divergent genetic backgrounds in multiple geographic locations has made this analysis, originally focused almost exclusively on the U.S. clone USA300, particularly challenging. Our previous analysis of virulence factors using isogenic gene deletion mutants in the Korean clone ST72^12^ to our knowledge is the only such analysis in a global CA-MRSA lineage performed as of yet; however, ST72 is of relatively limited geographic and clinical importance.

Here, we analyzed virulence and virulence determinants of a much more widespread CA-MRSA lineage, the predominant Asian CA-MRSA lineage ST59. As usually seen with CA-MRSA strains, CA-MRSA ST59 isolates had significantly more pronounced virulence in various animal infection models than the geographically matched HA-MRSA clones ST5 and ST239, which mirrors similar findings from our previous study that compared USA300 with common U.S. HA-MRSA clones^7^. Expression analyses showed increased expression of α-toxin, PSMs and the Agr regulator, which is in accordance with the notion that increased expression of these factors is characteristic for CA-MRSA^1^,^7^,^11^. Analysis of isogenic deletion mutants of those factors in *in-vitro* cytolysis assays demonstrated that PSMα peptides in ST59 CA-MRSA have a strong impact on lysis of neutrophils and erythrocytes, as previously shown for USA300^8^. α-toxin also showed a strong impact on neutrophil, but – as expected - not erythrocyte lysis. In animal skin, blood, and lung infection models, Agr, α-toxin, and PSMα had a strong effect on ST59 CA-MRSA pathogenesis.

Together with findings from previous studies that analyzed USA300 and ST72 gene deletion mutants in different animal models^8^,^9^,^12^,^26^,^27^, our results further emphasize that there are sometimes considerable differences in the relative contribution of even the core genome-encoded toxins to CA-MRSA virulence. For example, PSMα peptides had no significant impact on skin infection by ST72^12^, but a strong impact in the two clinically more important USA300^8^,^26^ and ST59 CA-MRSA. Furthermore, PSMα peptides were not as strong a contributor to virulence during severe lung infection in the USA300 background as PVL and α-toxin^27^, but here proved to be at least as important to the pathogenesis of lung infection as α-toxin. Currently, the reasons for these discrepancies between different strains are unknown. They do not seem to be due to expression differences, at least as far as what can be told from *in-vitro* expression levels. Differences may be due to differences in the animal models that were employed, *in-vivo* expression differences, and other, strain-specific toxins that may overshadow the effects of PSMα or α-toxin in a strain-dependent manner.

Overall, our findings strongly support the notion that Agr and the genome-encoded toxins α-toxin and PSMα are important general contributors to CA-MRSA virulence. They emphasize that drug development efforts that are underway to target α-toxin^28^,^29^,^30^ or the Agr regulator^31^,^32^ have high potential for the treatment of CA-MRSA infections and also call for including PSMα-targeting therapeutics in those endeavors. For the latter, blocking the recently discovered PSM transport system may bear great promise^33^,^34^.

## Methods

### Ethics statement

All animal experiments were performed following the Guide for the Care and Use of Laboratory Animals of the Chinese Association for Laboratory Animal Sciences (CALAS) with a protocol approved by the Committee on the Ethics of Animal Experiments of Renji Hospital (RJ-M-2014-0058).

### Bacterial strains, plasmids, and growth conditions

Bacteria were identified as staphylococci by classic microbiological methods. *S. aureus* strains were further categorized by VITEK2 automated systems (BioMérieux, France). All strains and plasmids used in this study are listed in Table 1. *Escherichia coli* was grown in Luria-Bertani medium. *S. aureus* was grown in tryptic soy broth (TSB) (Oxoid) with 0.25% glucose or on agar plates at 37 °C. Antibiotics were used at the following concentrations: ampicillin, 100 μg/ml; chloramphenicol, 10 μg/ml. Clinical U.S. ST59 SSTI CA-MRSA isolates were kindly provided by Binh Diep, University of California San Francisco^35^.

### Quantitative reverse-transcription (qRT) PCR

Total RNA was isolated using an RNeasy Mini Kit (Qiagen) from cells grown to late logarithmic growth phase (4 h) or stationary growth phase (8 h) in tryptic soy broth (TSB). Complementary DNA was synthesized from total RNA using the QuantiTect reverse transcription system (Qiagen) according to the manufacturer’s instructions. The resulting complementary DNA and negative control samples were amplified using the QuantiTect SYBR green PCR kit (Qiagen). Reactions were performed in a MicroAmp Optical 96-well reaction plate using a 7500 Sequence Detector (Applied Biosystems). All qRT-PCR experiments were performed in triplicate, with *gyrB* as control. Oligonucleotides are listed in Table 2.

### Lysis of erythrocytes by culture filtrates

Culture filtrates were collected from bacterial cultures grown for 18 h. Hemolytic activities were determined by incubating samples with human red blood cells (2% v/v in Dulbecco’s phosphate-buffered saline, DPBS) for 1 h at 37 °C. Hemolysis was determined by measuring the optical density at 540 nm using an ELISA reader. The assay was performed in triplicate.

### Neutrophil lysis by bacteria

Human neutrophils were isolated from heparinized venous blood of healthy volunteers with a standard method. Bacteria grown to mid-logarithmic growth phase and neutrophils were incubated at a 10:1 ratio. PBS with 0.1% Triton-X100 (100 μl) was used to determine 100% lysis. Lysis was measured using a lactate dehydrogenase (LDH) cytotoxicity detection kit according to the manufacturer’s protocol (Roche).

### Allelic gene replacement by homologous recombination and genetic complementation

For gene deletions in CA-MRSA ST59, a representative clinical isolate, RJ-2, was chosen, which was recovered from a 14 year-old female patient with severe SSTI. RJ-2 is *lukSF*-negative. The homologous recombination procedure using plasmid pKOR1 was performed as previously described^36^. Proper gene deletion was verified by analytical PCR and sequencing of the genomic DNA at the borders of the PCR–derived regions. Growth of the deletion mutants was similar to that of the wild-type strain. For genetic complementation of the *hla* mutation, the *hla* gene was PCR-amplified and cloned in the pTX_Δ_ plasmid via BamH1 and Nar1 restriction sites. Strains containing the empty plasmid pTX_Δ_16 were used as controls.

### Determination of PSM concentrations

PSM concentrations in culture filtrates of cultures grown for 8 h were analyzed by reversed-phase high-pressure chromatography/ion spray mass spectrometry as described^37^.

### Mouse infection models

The mouse skin infection model was performed essentially as described else where^8^. Briefly, outbred, immunocompetent hairless female mice between 4 and 6 weeks of age were used. Anesthetized mice were inoculated with 50 μl PBS containing ~10^7^ live *S. aureus* or PBS alone in the back by subcutaneous injection. Abscess lengths and widths were measured with a caliper and abscess areas were calculated using the formula length × width. Paraffin embedding and hematoxylin & eosin (H&E) staining were performed as previously described^38^.

For the lung infection model, female BALB/c mice were used at 4–6 weeks of age. 4 × 10^9^ CFU/40 μl *S. aureus* was pipetted into the nares of the anesthetized mice. 48 h after inoculation, all mice were euthanized. The lungs from each group of animals were excised, weighed and washed with PBS, and one lobe was fixed in 4% formalin for histological examination (H&E stain). The other lobe was homogenized in 0.5 ml of PBS, and *S. aureus* CFU/g lung tissue was determined by plating 100 μl homogenized lung tissue on TSB agar.

For the bacteremia model, we used BALB/c female mice 4–6 weeks of age. We injected each mouse with 100 μl PBS containing 10^7^ CFU live *S. aureus* into the retro-orbital vein. Control animals received sterile PBS only. After inoculation, mouse health and disease advancement were monitored every day. Mice were euthanized immediately if they showed signs of respiratory distress, mobility loss or inability to eat and drink. All animals were euthanized 4 days after injection, the livers and kidneys were excised, washed with PBS, and fixed in 4% formalin for histological examination (H&E stain). One kidney of each animal was homogenized in 0.5 ml of TSB, and the homogenized kidney tissue was diluted and plated on TSB agar for CFU determination.

### Statistics

Statistical analysis was performed using Graph Pad Prism 6.05. Data were analyzed using unpaired t tests to compare two different conditions and ANOVA for more conditions. All error bars show the standard error of the mean (SEM). All replicates are biological replicates.

## Additional Information

**How to cite this article**: Li, M. *et al*. Virulence determinants associated with the Asian community-associated methicillin-resistant *Staphylococcus aureus* lineage ST59. *Sci. Rep.* **6**, 27899; doi: 10.1038/srep27899 (2016).

## Figures and Tables

**Table 1:** Bacterial strains and plasmids used in this study.

Strain	Comment	Source
*S. aureus*		
RN4220	Derived from NCTC8325-4;r-m+	39
WT (RJ-2)	CA-MRSA ST59 clinical isolate RJ-2	This study
Δ*agr*	RJ-2 *agr* mutant; whole *agr* system deleted (from RNAIII to *agrA*)	This study
Δ*hla*	RJ-2 *hla* mutant	This study
Δ*hla(*pTX_Δ_16)	RJ-2 *hla* mutant with pTX_Δ_16	This study
Δ*hla(*pTX_Δ_*hla)*	RJ-2 *hla* mutant with pTX_Δ_*hla*	This study
Δ*psm*α	RJ-2 *psm*α mutant; *psm*α1-4 operon deleted.	This study
Δ*psm*α (pTX_Δ_16)	RJ-2 *psm*α mutant with pTX_Δ_16	This study
Δ*psm*α (pTX_Δ_*psm*α)	RJ-2 *psm*α mutant with pTX_Δ_*psm*α	This study
Plasmids		
pKOR1	cmR and ampR, temperature-sensitive (ts) vector for allelic replacement via lambda recombination and *ccdB* selection	36
pKOR1Δ*agr*	ts vector for allelic replacement of *agr* in ST59	This study
pKOR1Δ*hla*	ts vector for allelic replacement of *hla* in ST59	This study
pKOR1Δ*psm*α	ts vector for allelic replacement of *psm*α in ST59	This study
pTX_Δ_16	Control plasmid for the pTX_Δ_ plasmid series; lipase gene is deleted.	8
pTX_Δ_*psm*α	pTX_Δ_ plasmid containing the *psm*α*1-4* genes of *S. aureus*; *xylR* is deleted in that plasmid series for constitutive gene expression.	8
pTX_Δ_*hla*	pTX_Δ_ plasmid containing the *hla* gene of *S. aureus* ST59; *xylR* is deleted in that plasmid series for constitutive gene expression.	This study

**Table 2:** Oligonucleotides used in this study.

Oligonucleotide name	Sequence
*For isogenic deletion mutants*	
*agr-att1*	ggggacaagtttgtacaaaaaagcaggctaccctttcaattgtctgacg
*agr-rev1*	gatgaataattaattactttcattgtaaa
*agr-rev2*	agtaattaattattcatcacttacctatttaacgtttgtctaca
*agr-att2*	ggggaccactttgtacaagaaagctgggtgggatgcctttattggtg
*hla-att1*	ggggacaagtttgtacaaaaaagcaggctcccatgattagtgttctttta
*hla-rev1*	tttcatcatccttctattttt
*hla-rev2*	tagaaggatgatgaaaatgtaaattatttgttcatgtacaaa
*hla-att2*	ggggaccactttgtacaagaaagctgggtcgacgtaagaagaatcatatatatt
*psm*α*-att1*	ggggacaagtttgtacaaaaaagcaggctgccaaccacataaaaatgtc
*psm*α*-rev1*	taagattacctcctttgcttatgagtta
*psm*α*-rev2*	gcaaaggaggtaatcttatttaagcgaattgaatactt
*psm*α*-att2*	ggggaccactttgtacaagaaagctgggtccaaataatgtcgttcga
*For complementation*	
*hla*-BamH1-F	atcgggatccaattcaataaaggaggtgatgaaaatgaaaacacgtatagtc
*hla*-NarI-R	atcgggcgccttaatttgtcatttcttctttttcc
*For qRT-PCR*	
*gyrB*-f	caaatgatcacagcatttggtacag
*gyrB*-r	cggcatcagtcataatgacgat
*RNAIII*-f	atagcactgagtccaaggaaactaact
*RNAIII*-r	gccatcccaacttaataaccatgt
*hla-f*	aataactgtagcgaagtctggtgaaa
*hla*-r	gcagcagataacttccttgatcct
*psm*-f	tatcaaaagcttaatcgaacaattc
*psm*-r	ccccttcaaataagatgttcatatc
*For analytical PCR to detect ACME*	
*arcA-f*	gagccagaagtacgcgag
*arcA-r*	cacgtaacttgctagaacgag
*For analytical PCR to detect PVL*	
*lukS-f*	atcattaggtaaaatgtctggacatgatcca
*lukS-r*	gcatcaactgtattggatagcaaaagc
